# Supplementary material for: Integral equation solutions for the average run length for monitoring shifts in the mean of a generalized seasonal ARFIMAX(P, D, Q, r)s process running on a CUSUM control chart
Source: PLoS One. 2022 Feb 25;17(2):e0264283. doi: 10.1371/journal.pone.0264283 (PMC8880929; doi:10.1371/journal.pone.0264283)
Supplement: S1 File — (DOCX) [file pone.0264283.s001.docx]

**Appendices**

**Appendix A:** To partially prove the existence of the solutions for functional

***Proof.*** Let $\mathbf{C}([0, b])$ be a set of continuous functions on interval $[0, b].$ for any and define the sequence of iterations for $n\geq0,$ such that Subsequently, orbit is a Cauchy sequence of Moreover, since $ϒ$ is a contraction mapping, we arrive at

$$for \rho\in[0,1).$$

After repeating this argument *n* times, we obtain

Now, using the triangle inequality in this formula when $r$ is a positive integer enables us to derive

From the above, it follows that

Applying the property of the sum as a geometric series on $\rho$ gives

If $n\to\infty, \rho^{n}/(1-\rho)\to0.$ and letting $\varepsilon>0$ be arbitrary, then from above, it follows that

When is a Cauchy sequence, there exists unique point such that

By taking the limit $n\to\infty,$we obtain Since contraction maps are continuous, it follows that

Therefore is a fixed point on $M$such that , which completes the proof.

**Appendix B:** To partially prove the uniqueness of the solutions for functional

***Proof*:** Let and be two arbitrary functions on $\mathbf{C}([0, b]).$ and then complete metric space $\left((\mathbf{C}[0, h]), \left\| . \right\|_{\infty}\right).$ That is to say, a set of continuous functions of the ARL defined on $[0, b],$and $\mathbf{C}\left([0, b]\right)$become norm space if we define

for all functions where is a kernel function of the analytical IE for the ARL based on IEs obtained by using Theorem 4.1:

Hence, we obtain

where is a positive constant.

Thus,

The triangular inequality is used for the supremum norm as follows:

That is to say, $T : \mathbf{C}\left([0, b]\right)\to\mathbf{C}\left([0, b]\right)$ is a contraction mapping in complete metric space $\left(\left(\mathbf{C}[0, b]\right), \left\| . \right\|_{\infty}\right).$ Hence, by applying Theorem 4.2, the uniqueness of the ARL based on the analytical IE such that is confirmed. This completes the proof.

Therefore, the ARL based on the analytical IE for the CUSUM control chart for a long-memory $\mathrm{SARFIMAX}(P,D, Q, r)_{s}$ process exists and is unique.
