# Supplementary material for: Integral equation solutions for the average run length for monitoring shifts in the mean of a generalized seasonal ARFIMAX(P, D, Q, r)s process running on a CUSUM control chart
Source: PLoS One. 2022 Feb 25;17(2):e0264283. doi: 10.1371/journal.pone.0264283 (PMC8880929; doi:10.1371/journal.pone.0264283)
Supplement: S1 Table — (DOCX) [file pone.0264283.s002.docx]

|  | EUR | PTT |  | EUR | PTT |  | EUR | PTT |  | EUR | PTT |
| --- | --- | --- | --- | --- | --- | --- | --- | --- | --- | --- | --- |
| Jan-03 | 46.086 | 4.30 | Mar-07 | 46.763 | 20.80 | May-11 | 43.643 | 35.40 | Jul-15 | 38.458 | 32.60 |
| Feb-03 | 46.207 | 4.38 | Apr-07 | 47.456 | 22.00 | Jun-11 | 44.548 | 33.50 | Aug-15 | 40.182 | 26.80 |
| Mar-03 | 46.808 | 4.55 | May-07 | 46.594 | 25.40 | Jul-11 | 42.864 | 34.70 | Sep-15 | 40.651 | 24.00 |
| Apr-03 | 47.857 | 4.70 | Jun-07 | 46.752 | 27.00 | Aug-11 | 43.064 | 32.90 | Oct-15 | 39.180 | 27.50 |
| May-03 | 49.174 | 5.30 | Jul-07 | 46.177 | 31.40 | Sep-11 | 41.758 | 26.00 | Nov-15 | 37.821 | 25.60 |
| Jun-03 | 48.454 | 6.65 | Aug-07 | 46.764 | 30.60 | Oct-11 | 42.559 | 30.60 | Dec-15 | 39.130 | 24.40 |
| Jul-03 | 47.153 | 6.95 | Sep-07 | 48.910 | 33.60 | Nov-11 | 41.521 | 30.60 | Jan-16 | 38.708 | 23.60 |
| Aug-03 | 45.177 | 7.30 | Oct-07 | 49.217 | 41.60 | Dec-11 | 40.850 | 31.80 | Feb-16 | 38.746 | 26.00 |
| Sep-03 | 46.675 | 8.30 | Nov-07 | 49.532 | 38.00 | Jan-12 | 40.518 | 34.00 | Mar-16 | 39.955 | 28.00 |
| Oct-03 | 46.278 | 9.50 | Dec-07 | 49.146 | 37.60 | Feb-12 | 40.589 | 36.30 | Apr-16 | 39.981 | 30.40 |
| Nov-03 | 47.882 | 10.70 | Jan-08 | 49.083 | 32.60 | Mar-12 | 41.166 | 35.40 | May-16 | 39.819 | 30.10 |
| Dec-03 | 49.879 | 18.50 | Feb-08 | 47.785 | 34.20 | Apr-12 | 40.709 | 35.10 | Jun-16 | 39.002 | 31.30 |
| Jan-04 | 48.951 | 16.00 | Mar-08 | 49.658 | 31.60 | May-12 | 39.380 | 31.30 | Jul-16 | 38.852 | 33.00 |
| Feb-04 | 49.060 | 16.10 | Apr-08 | 49.436 | 33.40 | Jun-12 | 39.961 | 32.30 | Aug-16 | 38.644 | 34.90 |
| Mar-04 | 48.320 | 14.00 | May-08 | 50.533 | 33.60 | Jul-12 | 38.776 | 32.60 | Sep-16 | 38.883 | 33.90 |
| Apr-04 | 47.988 | 14.60 | Jun-08 | 52.672 | 30.20 | Aug-12 | 39.426 | 33.00 | Oct-16 | 38.466 | 34.60 |
| May-04 | 49.414 | 15.80 | Jul-08 | 52.291 | 25.00 | Sep-12 | 39.635 | 32.90 | Nov-16 | 37.810 | 34.90 |
| Jun-04 | 49.892 | 15.40 | Aug-08 | 50.222 | 26.60 | Oct-12 | 39.787 | 31.80 | Dec-16 | 37.700 | 37.20 |
| Jul-04 | 49.632 | 15.30 | Sep-08 | 47.797 | 22.80 | Nov-12 | 39.949 | 32.00 | Jan-17 | 37.901 | 40.40 |
| Aug-04 | 50.728 | 15.00 | Oct-08 | 44.629 | 15.90 | Dec-12 | 40.375 | 33.20 | Feb-17 | 36.956 | 39.70 |
| Sep-04 | 51.477 | 16.90 | Nov-08 | 45.043 | 14.70 | Jan-13 | 40.529 | 34.10 | Mar-17 | 36.593 | 38.70 |
| Oct-04 | 52.470 | 17.00 | Dec-08 | 48.659 | 17.50 | Feb-13 | 38.919 | 35.00 | Apr-17 | 37.693 | 38.90 |
| Nov-04 | 52.390 | 16.90 | Jan-09 | 44.692 | 16.00 | Mar-13 | 37.551 | 32.60 | May-17 | 38.273 | 39.30 |
| Dec-04 | 52.709 | 17.30 | Feb-09 | 45.856 | 15.60 | Apr-13 | 38.546 | 32.60 | Jun-17 | 38.768 | 37.00 |
| Jan-05 | 50.238 | 18.40 | Mar-09 | 47.027 | 15.25 | May-13 | 39.543 | 32.60 | Jul-17 | 39.434 | 38.80 |
| Feb-05 | 50.632 | 21.60 | Apr-09 | 46.656 | 18.95 | Jun-13 | 40.513 | 33.50 | Aug-17 | 39.517 | 39.90 |
| Mar-05 | 50.715 | 19.30 | May-09 | 48.562 | 22.00 | Jul-13 | 41.710 | 33.10 | Sep-17 | 39.376 | 40.80 |
| Apr-05 | 50.778 | 20.00 | Jun-09 | 47.805 | 23.40 | Aug-13 | 42.499 | 32.70 | Oct-17 | 38.700 | 42.00 |
| May-05 | 50.009 | 19.70 | Jul-09 | 48.486 | 23.90 | Sep-13 | 42.260 | 31.60 | Nov-17 | 38.867 | 41.20 |
| Jun-05 | 50.014 | 21.80 | Aug-09 | 48.759 | 24.40 | Oct-13 | 42.336 | 31.70 | Dec-17 | 39.066 | 44.00 |
| Jul-05 | 50.521 | 23.20 | Sep-09 | 48.928 | 26.20 | Nov-13 | 43.564 | 29.50 | Jan-18 | 38.940 | 49.20 |
| Aug-05 | 50.955 | 24.60 | Oct-09 | 49.239 | 24.00 | Dec-13 | 44.949 | 28.60 | Feb-18 | 38.399 | 56.80 |
| Sep-05 | 49.387 | 24.40 | Nov-09 | 49.887 | 22.40 | Jan-14 | 44.541 | 27.60 | Mar-18 | 38.422 | 55.20 |
| Oct-05 | 48.881 | 22.00 | Dec-09 | 47.767 | 24.60 | Feb-14 | 44.893 | 29.30 | Apr-18 | 38.114 | 56.50 |
| Nov-05 | 48.630 | 21.60 | Jan-10 | 45.999 | 22.30 | Mar-14 | 44.652 | 29.90 | May-18 | 37.485 | 52.25 |
| Dec-05 | 48.584 | 22.60 | Feb-10 | 45.041 | 23.20 | Apr-14 | 44.868 | 31.30 | Jun-18 | 38.596 | 48.00 |
| Jan-06 | 47.321 | 26.20 | Mar-10 | 43.713 | 26.20 | May-14 | 44.751 | 29.40 | Jul-18 | 38.816 | 51.25 |
| Feb-06 | 46.621 | 25.00 | Apr-10 | 43.012 | 25.70 | Jun-14 | 44.410 | 31.80 | Aug-18 | 38.035 | 52.50 |
| Mar-06 | 47.109 | 23.40 | May-10 | 40.017 | 24.60 | Jul-14 | 43.099 | 31.90 |  |  |  |
| Apr-06 | 47.427 | 25.80 | Jun-10 | 39.665 | 24.60 | Aug-14 | 41.945 | 32.10 |  |  |  |
| May-06 | 48.875 | 24.20 | Jul-10 | 42.067 | 25.40 | Sep-14 | 40.966 | 36.00 |  |  |  |
| Jun-06 | 48.752 | 22.60 | Aug-10 | 39.671 | 26.50 | Oct-14 | 40.830 | 36.80 |  |  |  |
| Jul-06 | 48.330 | 23.60 | Sep-10 | 41.376 | 29.70 | Nov-14 | 40.917 | 38.30 |  |  |  |
| Aug-06 | 48.114 | 23.60 | Oct-10 | 41.766 | 30.30 | Dec-14 | 39.816 | 32.40 |  |  |  |
| Sep-06 | 47.602 | 21.60 | Nov-10 | 39.180 | 30.90 | Jan-15 | 36.947 | 34.60 |  |  |  |
| Oct-06 | 46.824 | 22.20 | Dec-10 | 40.222 | 32.00 | Feb-15 | 36.229 | 34.30 |  |  |  |
| Nov-06 | 47.527 | 23.00 | Jan-11 | 42.312 | 33.50 | Mar-15 | 34.919 | 32.30 |  |  |  |
| Dec-06 | 47.714 | 21.00 | Feb-11 | 42.226 | 33.70 | Apr-15 | 37.063 | 35.60 |  |  |  |
| Jan-07 | 46.696 | 19.90 | Mar-11 | 42.862 | 35.40 | May-15 | 37.030 | 34.70 |  |  |  |
| Feb-07 | 46.989 | 20.80 | Apr-11 | 44.206 | 37.50 | Jun-15 | 37.645 | 35.90 |  |  |  |
