# Supplementary material for: Integral equation solutions for the average run length for monitoring shifts in the mean of a generalized seasonal ARFIMAX(P, D, Q, r)s process running on a CUSUM control chart
Source: PLoS One. 2022 Feb 25;17(2):e0264283. doi: 10.1371/journal.pone.0264283 (PMC8880929; doi:10.1371/journal.pone.0264283)
Supplement: Attachment — Submitted filename: Response to Reviewers.pdf [file pone.0264283.s005.pdf]

Reply to Reviewer's Comments on Manuscript Number: PONE-D-21-31542R1 - [EMID:918b38e0fabf7b8c]

Integral Equation Solutions for the Average Run Length for Monitoring Shifts in the Mean of a Generalized Seasonal ARFIMAX(P, D, Q, r) Process Running on a CUSUM Control Chart

The answer to reviewers comments for the revised manuscript.

The revised manuscripts are shown by using yellow highlight.

Reviewer comments	Revised manuscript
1. We note that your Data Availability Statement is currently as follows: "https://th.investing.com"<br><br>At this time we request that you please provide any additional instructions and/or direct access details (links, dataset titles, DOIs, accession numbers, or any other applicable persistent identifiers) that could be used by other researchers to locate the data underlying your study through the link provided.	The authors revised by reviewer suggestion.<br><br>I added the link to search locate the dataset provided for the monthly stock price data for PTT Public Company Ltd. Available from: https://th.investing.com/equities/ptt-historical-data And search EUR/THB exchange rate the dataset at: https://th.investing.com/currencies/eur-thb-historical-data with revised manuscript.
2. Based on the information you have provided us, we propose the following funding and competing interests statements for your approval:<br>Funding:<br>King Mongkut's University of Technology North Bangkok has received funding support from the National Science, Research and Innovation Fund (NSRF) (Grant No. KMUTNB-BasicR-64-20). The funders had no role in study design, data collection and analysis, decision to publish, or preparation of the manuscript.<br><br>Competing interests:<br>The authors have declared that no competing interests exist.<br><br>Can you please confirm the above statements are both complete and correct? With your approval we will update your competing interests and funding statements on your behalf.	The authors revised by reviewer suggestion.<br><br>Funding:<br>This work was supported by Grant No. KMUTNB-BasicR-64-20, National Science, Research and Innovation Fund (NSRF) via King Mongkut's University of Technology North Bangkok, to Wilasinee Peerajit. The funders had no role in study design, data collection and analysis, decision to publish, or preparation of the manuscript.<br><br>I confirmed that my statement is correct.
