# Supplementary material for: Integral equation solutions for the average run length for monitoring shifts in the mean of a generalized seasonal ARFIMAX(P, D, Q, r)s process running on a CUSUM control chart
Source: PLoS One. 2022 Feb 25;17(2):e0264283. doi: 10.1371/journal.pone.0264283 (PMC8880929; doi:10.1371/journal.pone.0264283)
Supplement: Attachment — Submitted filename: CommentFromReviewer.docx [file pone.0264283.s003.docx]

**Comments for Manuscript Number: PONE-D-21-31542**

Integral Equation Solutions for the Average Run Length for Monitoring Shifts in the

Mean of a Generalized Seasonal ARFIMAX(P, D, Q, r) Process Running on a CUSUM

Control Chart

Comment1:

What method do you apply to estimate numerical integral equation?. In abstract, you wrote Gauss-Legendre quadrature rules but in Definition 5: quadrature rules.

They are different.

Comment2:

repeat "IE"

Comment3:

Why do you use EUR/THB exchange rate to be the exogenous variable. Normally, Thai Baht is depended on the US dollar. You should also show the results of USD/THB as the exogenous variable.

Comment4:

Please insert the references about application of the time series to these fields.

Comment5:

It is wrong. $\xi_{t}$ is an error term as a random variable. The error term cannot be written with equal sign in term of a pdf function. Please correct it.

Comment6:

Norm C(M) or infinity norm, please check.

Comment7:

You cannot prove the existence and uniqueness for the numerical integral equation. It is for the analytical integral equation.
